# Seasonal Changes in the Peak-to-Sustained Power Profile of Elite Sprint Kayakers

**DOI:** 10.3390/sports14090414

**Published:** 2026-09-20

**Authors:** Tomáš Mika, Martin Čunát, Matylda Malá, Miroslava Nováková

**Affiliations:** 1 Sports Research Institute of the Czech Armed Forces, 160 00 Prague, Czech Republic; 2 Faculty of Physical Education and Sport, Charles University, 160 00 Prague, Czech Republic

**Keywords:** kayak sprint, Wingate test, periodization, anaerobic power, body composition, blood lactate

## Abstract

This study examined whether training periodization is associated with a systematic shift in the anaerobic performance profile of elite Czech sprint kayakers, assessed through seasonal (winter–summer) changes in Wingate-derived anaerobic performance, blood lactate response, and body composition analysis. Twenty-five sprint kayakers (10 women, 15 men) completed a 30-s upper-body Wingate test and anthropometric assessment during the winter preparatory period and again during the summer competition period. From winter to summer, peak power decreased by 37.3 ± 34.9 W (*p* < 0.001, dz = −1.07) and by 0.31 ± 0.43 W·kg^−1^ (*p* = 0.001, dz = −0.73), whereas mean power increased by 14.8 ± 24.1 W (*p* = 0.005, dz = 0.61) and by 0.30 ± 0.28 W·kg^−1^ (*p* < 0.001, dz = 1.07). Stroke cadence increased by 2.8 ± 3.7 rpm (*p* = 0.001), while body mass decreased by 1.3 ± 1.5 kg (*p* < 0.001) and skinfold-derived body fat decreased by 0.9 ± 1.5% (*p* = 0.006); peak blood lactate did not differ significantly between seasons (*p* = 0.228). The pattern was broadly similar in men and women, although the increase in absolute mean power did not reach significance in women (*p* = 0.087) despite a significant increase in relative mean power (*p* = 0.002). Peak power was lower and mean power was higher in the competitive phase than in the preparatory phase, indicating a shift in the anaerobic power profile toward sustained output. Because this study compared a single preparatory–competitive pair without a repeated macrocycle or a non-training control condition, this pattern is best interpreted as a phase-associated difference consistent with seasonal training emphasis, rather than as a demonstrated causal effect of training. This shift, coinciding with the transition from the winter preparatory to the summer competition phase, is the central finding of the study; monitoring an athlete’s position along this continuum may help coaches individualise periodization and interpret single-time-point Wingate results.

## 1. Introduction

Sprint kayaking is a sport characterized by high demands on both aerobic and anaerobic energy systems, with race distances ranging from 200 to 1000 m requiring different physiological and metabolic profiles [1,2,3]. While maximal oxygen uptake (VO2max) has long been considered an important determinant of performance, anaerobic capacity and power output are critical, particularly for shorter sprint distances [1,4,5]. In elite male kayakers, Wingate-derived relative peak power has recently been shown to discriminate between national- and international-level athletes and to correlate positively with post-exercise blood lactate concentration, underscoring its relevance as a performance marker in this population [5]. Determinants of performance may also differ between sexes, with distinct physiological predictors reported for female flat-water kayakers [6].

The Wingate anaerobic test has traditionally been used to assess anaerobic capacity, providing measures such as peak power, mean power, and fatigue index [7,8]. However, most applications of this test have been conducted within a single testing window, typically during the preparatory or competition phase, rather than continuously throughout the season. Consequently, there is limited knowledge on how Wingate-derived indicators of anaerobic performance fluctuate across different training periods in sprint kayakers.

In addition to performance outcomes, metabolic and physiological markers such as blood lactate concentration, acid–base balance, and body composition are essential for understanding training adaptations and fatigue tolerance [9,10,11]. Monitoring these markers across the training season can help identify how different training phases—such as preparation and competition—influence the balance between performance and recovery [12,13].

Previous studies in endurance and sprint sports have shown that seasonal training leads to fluctuations in both aerobic and anaerobic parameters, reflecting the specificity and intensity of the training load [13,14]. To our knowledge, no published study has reported longitudinal, Wingate-derived upper-body anaerobic indices—including peak power, mean power, and fatigue index—in elite sprint kayakers assessed across phases of the training macrocycle.

Therefore, the aim of this study was to examine whether, and how the anaerobic performance profile of elite sprint kayakers changes during training macrocycle—specifically, whether the balance between maximal (peak) and sustained (mean) power output shifts from the winter preparatory phase to the summer competition phase—assessed through the Wingate test together with selected physiological and metabolic markers. Understanding this profile shift may provide valuable information for athlete monitoring in high-performance kayaking, optimizing training strategies and periodization.

## 2. Materials and Methods

### 2.1. Participants

A total of 25 elite sprint kayakers (15 men, 10 women; age 21.7 ± 2.4 years, range 18–25) took part in this study, each completing one paired winter–summer assessment (25 paired observations, 50 individual test sessions). All athletes competed in K1, K2, and/or K4 boat categories and were members of national teams or departmental sports centres established for the support of top-level sport within the Czech state-sponsored elite-athlete system; according to the Participant Classification Framework of McKay et al. [15], the sample corresponded to Tier 4 (Elite/International Level). The competitive caliber of the sample during the study period is illustrated by international results achieved in 2025, including a silver medal in the women’s K4 500 m at the ICF Junior & U23 World Championships and multiple top-20 finishes at the ICF Canoe Sprint World Championships in Milan. All participants were injury-free at the time of testing and provided informed consent prior to data collection.

### 2.2. Anthropometric Measurements

All anthropometric assessments were conducted in the morning before laboratory testing. Stature was measured to the nearest 0.1 cm using a calibrated stadiometer (SECA 213, SECA GmbH & Co., Hamburg, Germany). Skinfold thickness was determined at ten anatomical sites (triceps, biceps, subscapular, chest, axilla, suprailiac, abdomen, front thigh, calf, and forearm) with a Harpenden caliper (Baty International, Burgess Hill, West Sussex, UK; constant pressure 10 g·mm^−2^) following standardized procedures, and converted to percentage body fat using the method of Pařízková, based on the sum of the ten skinfolds [16]; all skinfold measurements, at both testing occasions, were performed by a single anthropometrist with more than 40 years of experience in kinanthropometric assessment, to minimise inter-rater measurement error. This skinfold-derived value was obtained at both testing occasions and is the body-fat measure used in the paired seasonal comparison. Body mass and body composition variables, including fat mass, fat-free mass, and predicted muscle mass, were obtained by multi-frequency bioelectrical impedance analysis (Tanita MC-780 MA P, Tanita, Tokyo, Japan), a device with previously reported acceptable reliability and validity for tracking body-composition changes in adult populations, including athletes [10,11]; this bioelectrical-impedance assessment was performed once, at the winter occasion only, and is reported descriptively by sex in Table 1.

### 2.3. Wingate Anaerobic Test

Upper-body anaerobic performance was evaluated using a 30-s Wingate test performed on a kayak-specific ergometer (Cyclus2, RBM elektronik-automation GmbH, Leipzig, Germany), following the general methodological principles established for the test [7]. Power output was sampled at 2 Hz throughout the 30-s effort. Peak power (PP) was defined as the single highest recorded power sample, mean power (MP) as the arithmetic mean of all power samples recorded over the 30 s, and the fatigue index (FI) was calculated as (PP − LP)/t, where LP is the lowest recorded power sample and t is the time elapsed (in seconds) between the occurrence of PP and LP, yielding FI in W·s^−1^. Before testing, participants completed an approximately 10-min individualized warm-up; the test itself began once participants reached a cadence of 120 rpm (men) or 110 rpm (women), thresholds applied as an internal laboratory standard rather than a universal criterion. The applied braking resistance was set relative to body mass at 0.68 N·kg^−1^ for male and 0.56 N·kg^−1^ for female athletes, in accordance with normative recommendations for kayak-ergometer testing [17]. The ergometer underwent annual calibration, with manufacturer-verified power-measurement accuracy within ±2%. PP and MP were expressed both in absolute terms (W) and relative to body mass (W·kg^−1^). Both the winter and summer testing sessions were scheduled within a 2–3 week window preceding the principal competition or training block of the respective phase, so that each assessment reflected the athlete’s status close to the peak of that period.

### 2.4. Biochemical Analysis

Capillary blood samples were drawn from the non-dominant hand at 5 and 7 min post-exercise after the Wingate test, with the higher of the two lactate values recorded for subsequent data analysis. Lactate concentration was determined using an enzymatic biosensor (SensoStar GL30, DiaSys, Holzheim, Germany) according to the standard laboratory protocol [9].

### 2.5. Training Load Monitoring and Analysis

Training diaries were recorded using the national application Yarmill (Pošepný, Prague, Czech Republic), which is employed in canoe sprint for systematic monitoring of training load. Athletes reported all training sessions, including water-based, strength, and complementary activities, with details on duration and training modality. Training-diary entries across the cohort were reviewed by the coaching staff for completeness and internal consistency; missing or ambiguous information was clarified directly with the relevant athlete or coach. As an additional verification, complete, unaggregated weekly records from athletes representing different training groups and crews were examined in detail. Weekly training volume (h·wk^−1^) and its distribution across training modalities were calculated, in line with current consensus recommendations for athlete load monitoring [12]. On-water training intensity was classified according to the official training methodology of the Czech Canoe Union [18], which defines the following intensity zones: PV (aerobic/endurance pace), RV (anaerobic-threshold/tempo pace), R (race-specific pace), and TT (time-trial pace); these zone designations were used, alongside off-water strength and complementary training, to characterise the distribution of training modalities reported in Section 3.3. For statistical description, training data were aggregated into seasonal periods (winter/preparatory and summer/competition) to allow comparison of training-load characteristics across phases of the annual cycle.

### 2.6. Statistical Analysis

Winter and summer values for each Wingate and anthropometric variable were compared using paired-samples *t*-tests, with effect sizes expressed as Cohen’s dz (the mean paired difference divided by the standard deviation of the paired differences). Analyses were conducted for the full sample (25 paired observations) and separately for men (15 paired observations) and women (10 paired observations), giving 27 individual comparisons in total. Statistical significance was set at α = 0.05. To formally test whether the magnitude of the seasonal (time) effect differed between sexes, a mixed-design (time × sex) ANOVA was additionally performed for peak power, mean power, cadence, and fatigue index. Because the braking resistance applied during the Wingate test was recalculated from each athlete’s body mass at every testing occasion, a sensitivity analysis was performed to evaluate whether the between-season reduction in resistance could account for the observed change in peak power: the season-to-season change in braking resistance (Δresistance) was correlated with the corresponding change in peak power (ΔPP) across all athletes, and the peak-power comparison was additionally repeated in the subgroup of athletes with minimal body-mass change (|Δmass| ≤ 1.0 kg), in whom the associated change in resistance was negligible.

Because 27 comparisons were performed on a partly overlapping set of variables and sub-groups, raw *p*-values were adjusted for multiple comparisons using the Benjamini–Hochberg false discovery rate (FDR) procedure, applied across all 27 tests simultaneously. The FDR method was preferred over the more conservative Bonferroni correction because the analysis was considered exploratory (i.e., intended to characterise the overall seasonal pattern across related variables and sub-groups rather than to test a single confirmatory hypothesis), and Bonferroni correction is known to substantially inflate the false-negative rate in this setting. Both the uncorrected *p*-value and the FDR-adjusted *p*-value are reported in Table 2; effects for which the FDR-adjusted *p*-value exceeded 0.05 are interpreted with caution and are flagged explicitly in the text. Analyses were performed in Python 3 (pandas 2.x, SciPy); FDR adjustment followed the standard Benjamini–Hochberg step-up procedure.

Detailed segmental body-composition data (fat-free mass and fat mass by limb) were available for a single (winter) measurement occasion only; consequently, body composition is reported descriptively by sex in Table 1 rather than as a paired winter–summer comparison. Body fat percentage in Table 1 (BIA) and in Table 2 (skinfold-derived) reflect two different assessment methods obtained on different occasions and are not interchangeable; the skinfold-derived value was used for the paired seasonal comparison because it was the only body-fat measure available at both the winter and summer testing occasions.

## 3. Results

### 3.1. Body Composition

Detailed segmental body-composition data from the bioelectrical impedance analysis were available for all 10 female and 15 male athletes (Table 1). Men had greater fat-free mass, total body water, and predicted muscle mass, and lower relative body fat, than women. Because a matching summer segmental dataset was not available, these values are reported descriptively rather than as a seasonal comparison; the seasonal changes in overall body mass and fat percentage reported in Section 3.2 are, however, based on paired winter–summer measurements.

### 3.2. Wingate Anaerobic Performance

Athletes exhibited significant seasonal differences in body composition and Wingate test performance (Table 2). Body mass (78.4 ± 11.3 vs. 77.0 ± 11.7 kg, *p* < 0.001) and skinfold-derived body fat percentage (13.2 ± 4.4 vs. 12.3 ± 4.7%, *p* = 0.006) were significantly lower in summer than in winter.

**Figure 1:**
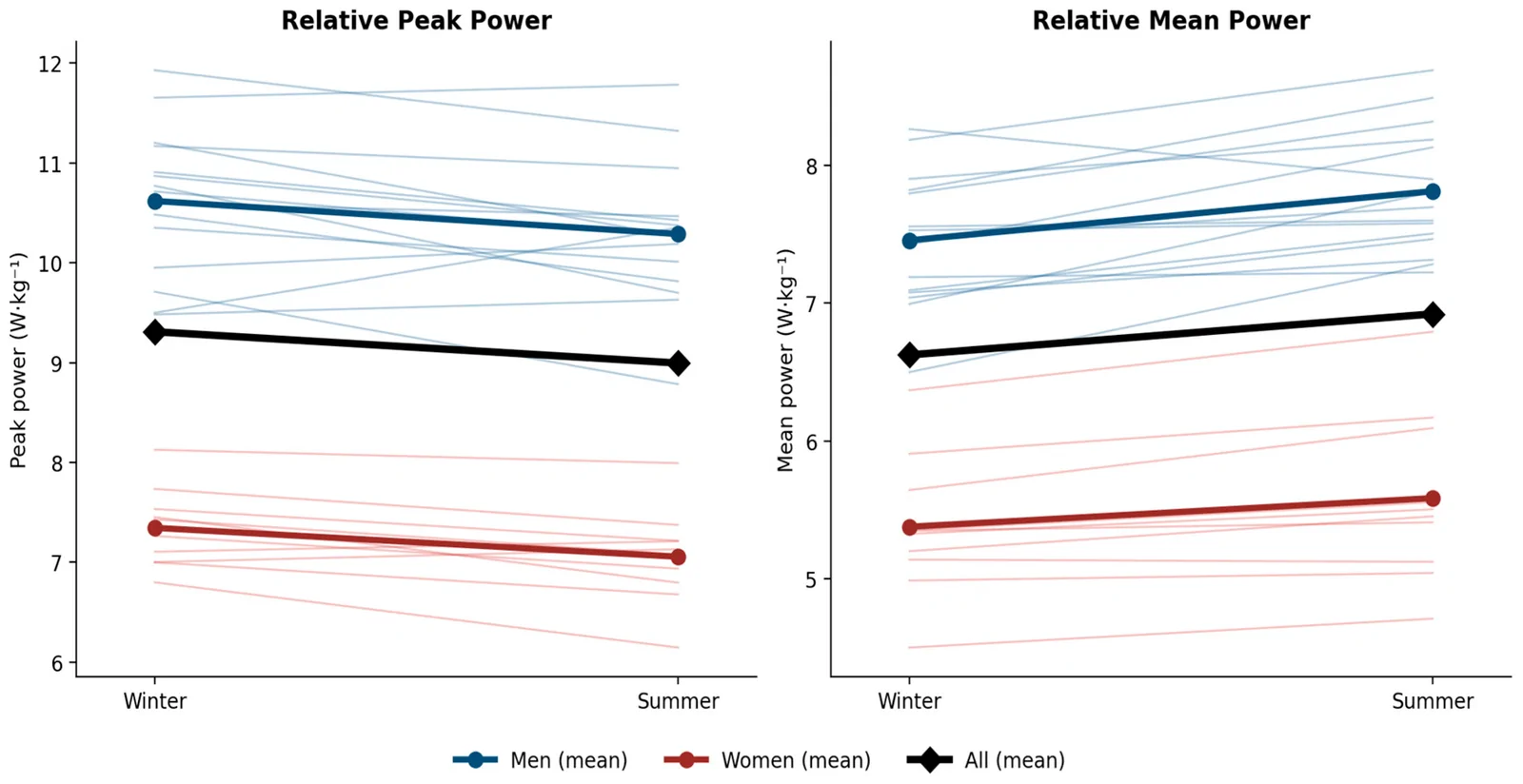
Individual seasonal change in relative peak power and relative mean power (W·kg^−1^). Thin lines represent individual athletes (blue = men, red = women); thick coloured lines with markers represent sex-specific group means, and the thick black line with diamond markers represents the overall (“All”) group mean. Most athletes show a downward shift in peak power and an upward shift in mean power from winter to summer.

Peak power decreased from winter to summer in both absolute (744.8 ± 226.1 vs. 707.5 ± 219.7 W, *p* < 0.001) and relative terms (9.31 ± 1.75 vs. 9.00 ± 1.73 W·kg^−1^, *p* = 0.001). In contrast, mean power increased from winter to summer in both absolute (527.1 ± 147.4 vs. 541.8 ± 156.8 W, *p* = 0.005) and relative terms (6.63 ± 1.15 vs. 6.92 ± 1.23 W·kg^−1^, *p* < 0.001). Cadence was significantly higher in summer (101.3 ± 10.0 vs. 104.0 ± 10.9 rpm, *p* = 0.001), and the fatigue index was significantly lower (13.8 ± 5.9 vs. 12.2 ± 4.8 W·s^−1^, *p* = 0.014). Peak blood lactate did not differ significantly between seasons (11.1 ± 2.6 vs. 10.7 ± 2.5 mmol·L^−1^, *p* = 0.228). The individual-athlete pattern underlying these group changes is illustrated in Figure 1, which shows relative peak power decrease and relative mean power increase from winter to summer in the great majority of individual athletes, consistent with the group-level statistics reported above.

When analysed separately by sex, the overall pattern was similar in men and women, with two notable exceptions. First, the increase in absolute mean power reached statistical significance in men (637.9 ± 59.3 vs. 657.9 ± 70.8 W, *p* = 0.018) but not in women (360.8 ± 35.9 vs. 367.7 ± 40.4 W, *p* = 0.087), whereas relative mean power increased significantly in both sexes (men: *p* < 0.001; women: *p* = 0.002). Second, the reduction in fatigue index reached nominal significance in men (*p* = 0.039) but not in women (*p* = 0.138). To formally test whether these apparent between-sex discrepancies reflected a genuine difference in the seasonal response, a mixed-design (time × sex) ANOVA was performed for each outcome. No significant time × sex interaction was found for peak power (F(1,23) = 0.89, *p* = 0.355), mean power (F(1,23) = 1.84, *p* = 0.188), cadence (F(1,23) = 0.03, *p* = 0.866), or fatigue index (F(1,23) = 1.25, *p* = 0.275), indicating that the magnitude of the seasonal shift did not differ significantly between men and women for any of these variables. The apparent between-sex discrepancies described above are therefore best interpreted as a consequence of the smaller female subgroup’s lower statistical power rather than as evidence of a true sex-specific response (see Limitations Section). Full results for all variables and both sexes are presented in Table 2.

It should be noted that the pooled (“All”) analysis reported above and in Table 2 averages across two sexes with distinct absolute power, body mass, and body-composition profiles; it is reported alongside, rather than instead of, the sex-specific analyses because the direction and pattern of the seasonal shift were consistent in men and women, but the pooled means themselves do not describe a physiologically homogeneous group and should be interpreted as a summary of a shared trend rather than a single representative athlete.

After adjusting for multiple comparisons (Benjamini–Hochberg FDR across all 27 tests), the great majority of effects remained statistically significant, including all peak power, mean power, and cadence comparisons for the full sample and for both sexes separately, as well as the reductions in body mass and body fat for the full sample and for men. Two effects that were nominally significant before correction did not survive FDR adjustment: the reduction in fatigue index in men (praw = 0.039, pFDR = 0.052) and the reduction in body mass in women (praw = 0.043, pFDR = 0.055). These two findings should therefore be interpreted as suggestive rather than confirmed. Peak lactate did not differ significantly between seasons either before or after correction (pFDR = 0.256–0.638).

A sensitivity analysis addressed whether the between-season change in braking resistance, which was recalculated from body mass at each testing occasion, could account for the observed reduction in peak power. The change in braking resistance did not correlate with the change in peak power across athletes (r = 0.14, *p* = 0.496), indicating that athletes with a larger reduction in resistance did not show a correspondingly larger decline in peak power. Furthermore, in the subgroup of athletes with minimal body-mass change between seasons (|Δmass| ≤ 1.0 kg, *n* = 10), in whom the associated change in braking resistance was negligible, peak power still declined significantly from winter to summer (*p* = 0.015, dz = 0.95). Together, these findings indicate that the seasonal reduction in peak power cannot be attributed to the recalculation of braking resistance from body mass alone.

### 3.3. Training Load Distribution

Analysis of the training diaries showed clear seasonal differences in the distribution of training modalities. In the winter period (January), average weekly training volume reached approximately 20 h, distributed as resistance training (6 h, 30%), ergometer training (6 h, 30%), running and cycling (5 h, 25%), swimming (2 h, 10%), and regeneration/physiotherapy (1 h, 5%); water-based training was not present. In the summer period (July), average weekly training volume was slightly lower (16–17 h) but with a markedly different structure: water-based training dominated (12 h, 70%), while resistance training (2 h, 12%) and ergometer training (2 h, 12%) decreased, running/cycling was reduced (1 h, 6%), and no swimming sessions were recorded. Thus, while the winter cycle emphasized general preparation (strength development, ergometer work, and complementary endurance), the summer phase was strongly oriented toward sport-specific performance on the water. These training-load data are reported at the group level; the training diaries were not analysed separately by sex, so an identical distribution of training modalities between men and women should not be assumed. Athletes in this cohort trained within shared coaching squads and crews (e.g., common K2/K4 crews training and competing under the same coaching staff); consequently, the principal structure and seasonal emphasis of training were substantially shared within each group, although individual adjustments remained possible. To examine the individual-level dynamics underlying the aggregated training description above, complete weekly records from athletes representing different training groups were analyzed in detail. Weekly training volume ranged from 4.0 to 23.25 h·wk^−1^ during winter and from 6.7 to 16.0 h·wk^−1^ during summer; the lowest values reflected identifiable circumstances, including injury recovery and competition/taper weeks, rather than random measurement variability. These individual records were consistent with the seasonal change in training composition described above at the group level and indicated that the observed pattern was present across different training groups rather than resulting from an isolated individual trajectory.

## 4. Discussion

The present findings show that peak power was lower, and mean power higher, in the competitive phase than in the preparatory phase—a pattern consistent with a shift in the anaerobic power profile toward sustained power output. As training phase and calendar season were fully confounded in this single-pair design, we interpret this pattern as associated with, rather than caused by, differences in training emphasis between phases (see also Limitations Section). This opposite-direction shift describes a coherent group effect along a single performance continuum, rather than an unrelated fluctuation in each variable independently, moving athletes from a more maximal-power-oriented profile in the preparatory phase toward a more sustained-power-oriented profile during the competition phase. This pattern occurred alongside a small but significant reduction in body mass and body fat from winter to summer and is consistent with the broader observation that anaerobic and body-composition variables can fluctuate meaningfully across a season, particularly when training volume and modality change substantially between phases [13,14,19]. Comparable seasonal changes in upper-body power have been reported in other upper-body-dominant sports: in elite Paralympic swimmers monitored across a competitive season, upper-body muscular power changed across successive assessment points, supporting the broader value of repeated, phase-based testing for individualizing upper-body training programmes [20].

A plausible physiological explanation is that the winter preparatory phase, which in this sample was dominated by resistance and ergometer training (Section 3.3), promotes gains in maximal neuromuscular power and, in combination with higher body mass, supports higher absolute peak power output. As athletes transition into the water-dominated summer phase, reduced body mass and a training stimulus more specific to sustained paddling effort appear to favour mean power output and a power-maintenance profile (reflected in higher cadence and a lower fatigue index) over single-effort maximal power. Because fatigue index is mathematically constrained by peak power, a lower fatigue index alongside a lower peak power does not by itself establish improved efficiency; it may instead partly reflect the lower starting peak against which the decline is calculated. In addition, as with the standard Wingate protocol, a non-negligible aerobic contribution to the 30-s effort has been described [21], a further factor relevant to interpreting the fatigue index and any seasonal change in aerobic conditioning. This interpretation is consistent with evidence that upper-body strength is an important determinant of kayak sprint performance [4] and that the metabolic profile of kayak sprint events combines a substantial anaerobic contribution with a non-trivial aerobic component even at short distances [1,2]. It is also consistent with reports that both anthropometric characteristics and body composition are meaningfully associated with force and power output of the kayak stroke [22], and that structured high-intensity interval training can drive measurable anaerobic adaptations in kayak athletes over a training block [19]. More broadly, the body-composition and power profile observed here is consistent with cross-sectional evidence that more advanced competitive levels in sprint kayaking are characterised by greater body mass, fat-free mass, and anaerobic power than less experienced counterparts [23], and with longitudinal DXA-based monitoring showing that senior kayakers’ body composition can change meaningfully across a training cycle, in both men and women [24].

Relative mean power increased from winter to summer in both sexes, whereas relative peak power decreased. Therefore, the seasonal shift cannot be explained solely by the reduction in body mass and instead appears to reflect a change in the shape of the 30-s power profile. From an applied perspective, this suggests that a single Wingate assessment should be interpreted in the context of the athlete’s current body mass and training phase rather than against fixed normative values [8], and that peak power and mean power may capture different, phase-dependent aspects of an athlete’s readiness (broadly analogous to a distinction between a more “power-oriented” profile in winter and a more “capacity-oriented” profile in summer). As the relative peak power (W·kg^−1^) values obtained during the Wingate test represent an important indicator distinguishing athletes competing at the international and national levels [5], the assessment of their seasonal changes may provide additional information for a more precise differentiation of athletes according to performance level.

The relatively stable peak lactate response across seasons, despite the changes in power output, may reflect a preserved glycolytic contribution to the 30-s effort regardless of training phase. This finding should also be considered in the context of the high performance level of the participants, who can be classified as elite athletes according to McKay et al. (2022) [15] and are therefore likely to have already developed a high degree of metabolic adaptation to the specific demands of sprint kayaking. Notably, the increase in mean power was not accompanied by a corresponding increase in peak lactate concentration, as might be expected [5] if the additional mechanical output were primarily supported by greater glycolytic energy production. Although the present data do not allow the relative contributions of individual energy systems to be quantified, this pattern may suggest that the higher mean power was partly supported by an increased aerobic contribution to the 30-s effort rather than by greater glycolytic involvement.

The apparent sex-specific pattern—a significant increase in relative but not absolute mean power in women—was not supported by a directly tested time × sex interaction, which was non-significant for mean power and all other Wingate outcomes (Section 3.2); it more likely reflects the smaller absolute range of power values and correspondingly lower statistical power in the female subgroup (*n* = 10) than a genuine sex difference in the seasonal response (see Limitations Section). This limited statistical power in women’s sprint kayak samples is a recurring feature of the literature in this population, where women’s squads are typically smaller than men’s and contemporary body-composition and performance data for women kayakers remain comparatively scarce [24].

The principal effects remained significant after FDR adjustment—the opposite-direction seasonal shift in peak versus mean power, the increase in cadence, and the reduction in body mass and body fat all remained statistically robust after adjusting for multiple comparisons using the Benjamini–Hochberg FDR procedure. Only two secondary, smaller-magnitude effects (the reduction in fatigue index in men and the reduction in body mass in women) fell below the corrected significance threshold and are therefore presented as suggestive rather than confirmed findings. This reduces the likelihood that the principal statistical findings resulted solely from multiple testing, although FDR adjustment addresses multiple comparisons and not confounding or measurement error.

### Limitations

Several limitations should be considered. This was an observational, single-season study without a non-training control condition, so the changes observed cannot be attributed to training alone. Detailed segmental body-composition data were available for a single time point only, preventing a paired seasonal comparison for these variables. The female subgroup was small (*n* = 10); although a formal mixed-design time × sex interaction test found no significant sex difference in the magnitude of the seasonal change for any outcome (Section 3.2), this limits statistical power to detect a genuine sex-specific effect if one exists.

Because braking resistance was recalculated from body mass at each testing occasion, the lower summer resistive load could in principle have contributed mechanically to the higher cadence and lower peak power observed in summer. A sensitivity analysis (Section 3.2) found no correlation between the change in braking resistance and the change in peak power, and peak power still declined significantly in the subgroup with minimal body-mass change, making a purely mechanical explanation unlikely, although it cannot be entirely excluded; the same consideration applies to relative power measures.

Training volume and intensity in the microcycles immediately preceding each test could not be systematically quantified for all athletes. However, the bidirectional pattern observed—lower peak power but higher mean power in summer—is not consistent with a simple residual-fatigue or freshness effect, which would be expected to shift all outcomes in the same direction. Full-cohort dispersion statistics for weekly training load were not calculated, so reported training characteristics describe the cohort’s shared periodization structure rather than individual variability. Because participants were Tier 4 (Elite/International Level) athletes [15] embedded within national-team calendars, a more powerful design (e.g., repeated macrocycles, a return-to-baseline measurement, or a non-training control group) was not logistically feasible.

Finally, findings are limited to Czech elite sprint kayakers within a single national system and season, which may limit generalizability. Test–retest reliability for the Wingate-derived indices was not established; the winter and summer assessments, separated by 5–8 months, cannot serve as a short-interval reliability pair, and a dedicated reliability sub-study was outside the present scope—future work should incorporate this alongside any seasonal comparison.

## 5. Conclusions

In conclusion, elite sprint kayakers exhibited lower peak power and higher mean power in the competitive phase than in the preparatory phase, consistent with a phase-associated shift in the anaerobic power profile toward sustained power output. This pattern might be associated with differences in training emphasis between seasonal training phases. In particular, it suggests that a single Wingate test provides only a phase-specific snapshot of anaerobic performance, and that coaches and sport scientists should interpret Wingate results relative to the athlete’s current training phase and body mass rather than against a single fixed reference point. Repeated seasonal testing, combined with training-load monitoring, may offer a more complete picture of how an athlete’s performance profile evolves across the training year. What anaerobic profile is favourable for the competition remains to be elucidated.

## Figures and Tables

**Table 1:** Descriptive body composition by sex (winter measurement occasion).

Variable	Women (*n* = 10), M ± SD	Men (*n* = 15), M ± SD
Body mass (kg)	67.5 ± 7.5	85.6 ± 6.5
Height (cm)	168.1 ± 6.3	185.2 ± 7.2
BMI (kg·m^−2^)	23.8 ± 1.6	25.0 ± 1.4
Body fat, BIA (%)	21.7 ± 4.9	11.0 ± 3.4
Fat-free mass (kg)	52.6 ± 4.3	76.2 ± 6.2
Total body water (kg)	38.8 ± 3.7	54.7 ± 4.4
Predicted muscle mass (kg)	50.0 ± 4.1	72.4 ± 6.0

**Table 2:** Seasonal (winter vs. summer) comparison of Wingate anaerobic performance, blood lactate, and body composition (paired-samples *t*-tests).

Variable	Group	*n*	Winter (M ± SD)	Summer (M ± SD)	Change (Summer–Winter)	95% CI	*p*	*p* (FDR)	dz
Peak power (W)	All	25	744.8 ± 226.1	707.5 ± 219.7	−37.3	−51.7 to −22.9	**<0.001**	**<0.001**	−1.07
	Men	15	910.3 ± 107.6	867.7 ± 107.4	−42.7	−65.0 to −20.4	**0.001**	**0.005**	−1.06
	Women	10	496.4 ± 63.6	467.2 ± 62.4	−29.2	−46.8 to −11.6	**0.005**	**0.011**	−1.19
Peak power (W·kg^−1^)	All	25	9.31 ± 1.75	9.00 ± 1.73	−0.31	−0.49 to −0.14	**0.001**	**0.005**	−0.73
	Men	15	10.62 ± 0.73	10.29 ± 0.71	−0.33	−0.62 to −0.04	**0.027**	**0.038**	−0.64
	Women	10	7.35 ± 0.40	7.06 ± 0.48	−0.29	−0.48 to −0.10	**0.007**	**0.014**	−1.09
Mean power (W)	All	25	527.1 ± 147.4	541.8 ± 156.8	+14.8	4.8 to 24.7	**0.005**	**0.012**	0.61
	Men	15	637.9 ± 59.3	657.9 ± 70.8	+20.0	4.0 to 36.0	**0.018**	**0.029**	0.69
	Women	10	360.8 ± 35.9	367.7 ± 40.4	+6.9	−1.2 to 15.0	0.087	0.107	0.61
Mean power (W·kg^−1^)	All	25	6.63 ± 1.15	6.92 ± 1.23	+0.30	0.18 to 0.41	**<0.001**	**<0.001**	1.07
	Men	15	7.46 ± 0.49	7.81 ± 0.46	+0.36	0.17 to 0.54	**0.001**	**0.005**	1.08
	Women	10	5.38 ± 0.51	5.59 ± 0.61	+0.21	0.10 to 0.32	**0.002**	**0.006**	1.37
Cadence (rpm)	All	25	101.3 ± 10.0	104.0 ± 10.8	+2.8	1.2 to 4.3	**0.001**	**0.005**	0.74
	Men	15	107.1 ± 5.7	109.9 ± 6.0	+2.9	0.4 to 5.3	**0.024**	**0.036**	0.65
	Women	10	92.6 ± 8.9	95.2 ± 10.6	+2.6	0.7 to 4.5	**0.014**	**0.024**	0.96
Fatigue index (W·s^−1^)	All	25	13.8 ± 5.9	12.2 ± 4.8	−1.5	−2.75 to −0.34	**0.014**	**0.024**	−0.53
	Men	15	17.2 ± 5.0	15.1 ± 3.6	−2.1	−4.02 to −0.12	0.039	0.052	−0.59
	Women	10	8.6 ± 2.0	7.9 ± 2.5	−0.8	−1.79 to 0.29	0.138	0.162	−0.51
Body mass (kg)	All	25	78.4 ± 11.3	77.0 ± 11.7	−1.3	−1.94 to −0.72	**<0.001**	**0.001**	−0.90
	Men	15	85.6 ± 6.5	84.2 ± 7.4	−1.4	−2.17 to −0.62	**0.002**	**0.006**	−0.99
	Women	10	67.5 ± 7.5	66.3 ± 8.0	−1.2	−2.43 to −0.05	0.043	0.055	−0.74
Body fat, skinfold (%)	All	25	13.2 ± 4.4	12.3 ± 4.7	−0.9	−1.51 to −0.28	**0.006**	**0.013**	−0.60
	Men	15	11.0 ± 2.4	10.0 ± 2.3	−1.0	−1.60 to −0.40	**0.003**	**0.008**	−0.93
	Women	10	16.4 ± 4.8	15.7 ± 5.4	−0.7	−2.16 to 0.70	0.279	0.290	−0.36
Peak lactate (mmol·L^−1^)	All	25	11.1 ± 2.6	10.7 ± 2.5	−0.4	−0.94 to 0.23	0.228	0.256	−0.25
	Men	15	12.4 ± 1.9	11.9 ± 1.8	−0.5	−1.37 to 0.42	0.278	0.290	−0.29
	Women	10	9.1 ± 2.2	9.0 ± 2.3	−0.2	−0.97 to 0.63	0.638	0.638	−0.15

Note: *p* = uncorrected paired-samples *t*-test *p*-value; *p* (FDR) = Benjamini–Hochberg false-discovery-rate-adjusted *p*-value across all 27 comparisons in this table; dz = Cohen’s dz for paired samples (mean difference/SD of differences). Negative dz indicates lower summer values. Bold interpretation should rely on *p* (FDR). 95% CI represents the confidence interval for the mean paired difference.

## Data Availability

The data presented in this study are available on request from the corresponding author. The data are not publicly available due to privacy restrictions relating to identifiable athlete health and performance records.
